# Surgical Subspecialty and Parathyroidectomy Outcomes: A National Analysis

**DOI:** 10.1007/s12070-025-05436-1

**Published:** 2025-04-02

**Authors:** Owais M. Aftab, Roshan V. Patel, Avneet Randhawa, Karandeep Randhawa, Imran Khawaja, Hamza Khan, David Mothy, Jean Anderson Eloy, Christina H. Fang

**Affiliations:** 1 Department of Otolaryngology– Head and Neck Surgery, Rutgers New Jersey Medical School, 90 Bergen, Street, Suite 8100, Newark, NJ 07103 USA; 2 Center for Skull Base and Pituitary Surgery, Neurological Institute of New Jersey, Rutgers New Jersey Medical School, 140 Bergen Street, Newark, NJ 07103 USA; 3 Department of Neurological Surgery, Rutgers New Jersey Medical School, 90 Bergen Street Newark, Newark, NJ 07101 − 1709 USA; 4 Department of Ophthalmology and Visual Science, Rutgers New Jersey Medical School, 90 Bergen Street Newark, Newark, NJ 07103 USA; 5 Department of Otolaryngology and Facial Plastic Surgery, Saint Barnabas Medical Center– RWJ Barnabas Health, 94 Old Short Hills Rd, Livingston, NJ 07039 USA; 6 Department of Otorhinolaryngology– Head and Neck Surgery, Montefiore Medical Center, The University Hospital of Albert Einstein College of Medicine, 3400 Bainbridge Avenue, Medical Arts Pavilion, 3rd Floor, Bronx, NY 10467 USA

**Keywords:** Parathyroidectomy, General Surgery, Otolaryngology, Outcomes, NSQIP

## Abstract

**Aims:**

Parathyroidectomy (PT) is commonly performed for hyperparathyroidism. We investigated the association between surgical subspecialty and adverse outcomes in patients undergoing PT.

**Materials and methods:**

This retrospective cohort analysis utilized the 2005–2018 National Surgery Quality Improvement Program (NSQIP) database. Current Procedural Terminology (CPT) codes were used to identify cases with a primary procedure of PT. Demographics, comorbidities, and complication incidences were compared between patients undergoing surgery by general surgeons or otolaryngologists using chi-square analyses. The independent effect of surgical subspecialty on adverse outcomes was analyzed using binary logistic regression.

**Results:**

49,667 (86.7%) PT performed by general surgeons and 7,595 (13.3%) by otolaryngologists were identified from 2005 to 2018. Chi-square analysis indicated that general surgery patients had lower incidences of obesity (42.0% vs. 44.6%; *p* < 0.001) and higher incidences of diabetes mellitus (8.1% vs. 5.8%; *p* < 0.001). Demographic characteristics that significantly differed between cohorts included race (*p* < 0.001) and age (*p* < 0.001). Unadjusted analyses indicated that otolaryngologist-performed PT had lower incidences of unplanned reoperation (0.9% vs. 1.1%; *p* = 0.048) and unplanned readmission (2.9% vs. 3.6%; *p* = 0.009). After adjusting for confounders, logistic regression analyses indicated that otolaryngologist-performed PT had increased odds of prolonged operation time (OR 1.605; 95% CI 1.475–1.746; *p* < 0.001). Significant differences in mortality, medical complications, and surgical complications were not found.

**Conclusion:**

Surgical subspecialty is associated with PT operative time but is not associated with perioperative complications.

**Supplementary Information:**

The online version contains supplementary material available at 10.1007/s12070-025-05436-1.

## Introduction

Primary hyperparathyroidism (PHPT) is among the most common endocrine disorders in the United States and frequently presents asymptomatically, which is typical of developed nations [1, 2]. PHPT can occur in isolation, as the result of an adenoma of a single gland (80% of cases), but can also be a part of rarer syndromes or a multiple glandular disorder [3]. While typically discovered when asymptomatic, hyperparathyroidism can progress into a symptomatic disease with bone loss or kidney stones [1]. PHPT has been reported with the highest incidence in post-menopausal women (74%) and with a higher incidence in Black Americans than White Americans [4, 5]. Parathyroidectomy is indicated for symptomatic PHPT and, in some cases, subclinical PHPT (in cases of end-organ effects) [6].

Parathyroidectomy can be performed by both general or endocrine surgeons and otolaryngologists [7]. While prior literature has compared outcomes by provider-type in procedures overlapping between plastic surgery and otolaryngology (ENT) as well as general surgery and otolaryngologists with variable findings, relatively fewer studies have evaluated the influence of surgical subspecialty on outcomes in parathyroidectomy [8, 9]. As such, this study aims to utilize the National Surgery Quality Improvement Program (NSQIP) to evaluate the impact of surgical subspecialty, specifically general surgery vs. otolaryngology on outcomes following parathyroidectomy.

## Methods

### Database

This retrospective cohort analysis utilized the American College of Surgeons National Surgery Quality Improvement Program (NSQIP), a nationally validated, risk-adjusted database that includes information regarding demographic variables, comorbidities, preoperative laboratory values, and postoperative complications up to 30 days after surgery. The NSQIP collects information on over 1 million cases per year from 680 different institutions nationally and can play a role in the reduction of surgical complications, and by extension, costs [10, 11]. As NSQIP contains de-identified data, this study was deemed as exempt by the Rutgers New Jersey Medical School Institutional Review Board, Newark, New Jersey.

### Study Population

Cases with a primary Current Procedural Terminology (CPT) codes of parathyroidectomy with exploration of parathyroid(s) (60500), parathyroidectomy with re-exploration (60502), or parathyroidectomy with mediastinal exploration with a sternal split or transthoracic approach (60505) were identified in accordance with other retrospective analyses of parathyroidectomy outcomes [12]. Cases with missing data were excluded from this analysis.

### Study Variables

Postoperative outcomes evaluated in this study include progressive renal insufficiency, transfusion, stroke/cardiovascular accident, cardiac arrest, myocardial infarction, superficial incisional surgical site infection (SSI), organ space SSI, urinary tract infection, septic shock, sepsis, pneumonia, wound disruption, pulmonary embolism, acute renal failure, deep incisional SSI, unplanned intubation, deep venous thrombosis, any surgical complication, any medical complication, mortality, prolonged length of hospital stay (LOS) (defined as an LOS greater than the 90th percentile of selected cases), prolonged operative time (defined as an operation time greater than the 90th percentile of selected cases), unplanned reoperation, and unplanned readmission.

The primary exposure variable was surgery performed by either a general surgeon or otolaryngologist. To adjust for potential confounding factors, demographic variables like gender, age cohort (< 35, 35–49, 50–65, 65 + years), race (White, Black, Asian, Hawaiian/Pacific, Native American, Unknown), and Hispanic ethnicity were evaluated in addition to preoperative comorbidities including obesity, diabetes, smoking, dyspnea, poor functional status (defined as a partially or totally dependent health status prior to surgery), ventilator dependence, chronic obstructive pulmonary disease, ascites, congestive heart failure, hypertension, renal failure, dialysis, disseminated cancer, open wound(s), steroid use, weight loss, bleeding disorders, preoperative blood transfusion, and preoperative systemic sepsis.

### Statistical Analysis

Demographics, comorbidities, and complications were compared between patients undergoing surgery by general surgeons or otolaryngologists using univariate Pearson chi-square analyses and Mann-Whitney U-tests as appropriate. Significantly differing preoperative factors were adjusted for using multivariate, binary logistic regression to isolate the independent influence of surgical subspecialty on postoperative outcomes. The alpha level was set at *p* < 0.05. All statistical analysis was performed using SPSS Statistics Version 25.0 (IBM Corporation, Armonk, NY).

## Results

49,667 (86.7%) parathyroidectomy cases performed by general surgeons and 7,595 cases (13.3%) by otolaryngologists were identified from 2005 to 2018. Chi-square analysis indicated that these cohorts of patients were 74.8% and 75.1% female, respectively (Table 1), with no significant gender differences. However, chi-square analysis indicated that patients significantly differed in age, with 25.8% of general surgery patients and 28.0% of otolaryngologist patients being 65 + years (*p* < 0.001). Cohorts significantly differed in race, with 76.5% of general surgery patients and 71.9% of otolaryngologist patients being White (*p* < 0.001). Patients operated on by otolaryngologists were significantly more often Hispanic (7.6% vs. 5.4%, *p* < 0.001).

**Table 1:** Demographics and comorbidities of patients undergoing parathyroidectomy according to surgical subspecialty

	General Surgery	Otolaryngology	*p*-value
Gender			0.619
Female	74.8%	75.1%	
Male	25.2%	24.9%	
Age cohorts, years			**< 0.001**
< 35	4.8%	4.9%	
35–49	15.0%	13.9%	
50–65	54.3%	53.1%	
65+	25.8%	28.0%	
Race			**< 0.001**
White	76.5%	71.9%	
Black	15.2%	12.6%	
Asian	2.0%	2.5%	
Hawaiian/Pacific	0.2%	0.3%	
Native American	0.3%	0.4%	
Unknown	5.9%	12.2%	
Hispanic ethnicity	5.4%	7.6%	**< 0.001**
Obese	42.0%	44.6%	**< 0.001**
Diabetic	8.1%	5.8%	**< 0.001**
Smoker	11.5%	10.9%	0.113
Dyspnea	7.1%	5.0%	**< 0.001**
Poor functional status	1.4%	1.2%	0.175
Ventilator dependence	0.0%	0.0%	1.000
Chronic obstructive pulmonary disease	2.5%	2.9%	0.093
Ascites	0.0%	0.1%	0.238
Congestive heart failure	0.5%	0.4%	0.323
Hypertension	57.8%	56.1%	**0.005**
Renal failure	0.5%	0.5%	0.597
Dialysis	6.9%	4.6%	**< 0.001**
Disseminated cancer	0.3%	0.3%	0.920
Open wound	0.6%	0.5%	0.361
Steroid use	4.0%	3.1%	**< 0.001**
Weight loss	0.6%	0.5%	0.157
Bleeding disorder	2.2%	1.8%	**0.035**
Preoperative blood transfusion	0.1%	0.1%	0.143
Preoperative Systemic sepsis	0.5%	0.3%	**0.014**

Furthermore, additional chi-square analysis of comorbidities indicated that patients operated on by general surgeons more often had obesity (42.0% vs. 44.6%; *p* < 0.001), diabetes mellitus (8.1% vs. 5.8%; *p* < 0.001), dyspnea on exertion (7.1% vs. 5.0%; *p* < 0.001), hypertension (57.8% vs. 56.1%; *p* = 0.005), dialysis (6.9% vs. 4.6%; *p* < 0.001), steroid use (4.0% vs. 3.1%; *p* < 0.001), bleeding disorders (2.2% vs. 1.8%; *p* = 0.035), and preoperative systemic sepsis (0.5% vs. 0.3%; *p* = 0.014).

Chi-square analysis indicated that cases of ENT-performed parathyroidectomy had less postoperative complications, including septic shock (0.0% vs. 0.1%; *p* = 0.012), acute renal failure (0.0% vs. 0.1%; *p* = 0.011), unplanned reoperation (0.9% vs. 1.1%; *p* = 0.048) and unplanned readmission (2.9% vs. 3.6%; *p* = 0.009) (Table 2). A larger proportion of ENT cases had prolonged operative time (12.7% vs. 9.7%; *p* < 0.001) but a lower proportion had prolonged LOS (10.7% vs. 11.7%; *p* = 0.008). On unadjusted Mann-Whitney U-tests of operative time and LOS, ENT-performed PT cases had a significantly longer operative time (99.88 min vs. 91.52 min; *p* < 0.001) with a significantly shorter LOS (1.00 days vs. 1.18 days; *p* < 0.001). No other significant differences in complications like SSI, any surgical complication, any medical complication, or morbidity were found in unadjusted analysis.

**Table 2:** Unadjusted analysis of complications in patients undergoing parathyroidectomy according to surgical subspecialty. Chi square analysis and Mann-Whitney U were used as appropriate

	General Surgery	Otolaryngology		*p*-value
Ventilation > 48 h	N/A	N/A	N/A*	
Progressive renal insufficiency	0.1%	0.1%	0.814	
Transfusion	0.1%	0.2%	0.614	
Stroke/cardiovascular accident	0.1%	0.1%	0.327	
Cardiac arrest	0.1%	0.0%	0.200	
Myocardial infraction	0.1%	0.1%	0.461	
Superficial incisional SSI	0.2%	0.2%	0.499	
Organ space SSI	0.0%	0.0%	0.618	
Urinary tract infection	0.4%	0.4%	0.703	
Septic shock	0.1%	0.0%	**0.012**	
Sepsis	0.2%	0.2%	0.645	
Pneumonia	0.2%	0.1%	0.103	
Wound disruption	0.0%	0.0%	1.000	
Pulmonary embolism	0.1%	0.1%	0.803	
Acute renal failure	0.1%	0.0%	**0.011**	
Deep incisional SSI	0.0%	0.1%	0.153	
Unplanned intubation	0.3%	0.2%	0.131	
Deep venous thrombosis	0.1%	0.1%	1.000	
Any surgical complication	0.4%	0.4%	0.435	
Any medical complication	1.4%	1.2%	0.165	
Any complication	1.7%	1.6%	0.498	
Death	0.2%	0.1%	0.877	
Prolonged length of stay	11.7%	10.7%	**0.008**	
Prolonged operative time	9.7%	12.7%	**< 0.001**	
Unplanned reoperation	1.1%	0.9%	**0.048**	
Unplanned readmission	3.6%	2.9%	**0.009**	
Length of Stay (Median, IQR)	1.0 (0.0, 1.0) days	0.0 (0.0, 1.0) days	**< 0.001**	
Operative time (Median, IQR)	79.0 (58.0, 112.0) min	87.0 (63.0, 123.0) min	**< 0.001**	

SSI– surgical site infection

*No cases were reported in NSQIP for this complication

After adjusting for confounders, logistic regression analyses indicated that ENT-performed parathyroidectomy had increased odds of prolonged operative time (OR 1.605; 95% CI 1.475–1.746; *p* < 0.001) (Table 3). No other significant differences in adverse outcomes were found, including any surgical complications (OR 1.179; 95% CI 0.751–1.850; *p* = 0.474), any medical complications (OR 0.825; 95% CI 0.619–1.099; *p* = 0.188), any complications (OR 0.929; 95% CI 0.727–1.187; *p* = 0.556), death (OR 0.828; 95% CI 0.353–1.940; *p* = 0.663); prolonged LOS (OR 1.001; 95% CI 0.890–1.126; *p* = 0.986), unplanned reoperation (OR 0.871; 95% CI 0.641–1.183; *p* = 0.376), or unplanned readmission (OR 0.851; 95% CI 0.688–1.051; *p* = 0.134).

**Table 3:** Binary logistic regression analysis of adverse outcomes after parathyroidectomy performed by otolaryngologists vs. general surgeons. To adjust for confounders, regression included patient demographics and significantly differing comorbidities as covariates

Outcome	Odds Ratio	95% Confidence Interval	*p*-value
Progressive renal insufficiency	0.823	0.247–2.743	0.751
Transfusion	0.927	0.407–2.111	0.857
Stroke/cardiovascular accident	1.331	0.453–3.910	0.603
Cardiac arrest	0.492	0.117–2.064	0.332
Myocardial infraction	0.792	0.278–2.253	0.661
Superficial incisional SSI	1.413	0.773–2.582	0.261
Organ space SSI	0.000	0.000– NA*	0.979
Urinary tract infection	0.936	0.584–1.500	0.784
Septic shock	0.000	0.000– NA*	0.978
Sepsis	1.713	0.824–3.562	0.150
Pneumonia	0.401	0.156–1.102	0.077
Wound disruption	0.880	0.109–7.122	0.904
Pulmonary embolism	1.198	0.406–3.540	0.743
Acute renal failure	0.000	0.000– NA*	0.977
Deep incisional SSI	2.676	0.697–10.273	0.152
Unplanned intubation	0.649	0.327–1.291	0.218
Deep venous thrombosis	0.662	0.236–1.858	0.433
Any surgical complication	1.179	0.751–1.850	0.474
Any medical complication	0.825	0.619–1.099	0.188
Any complication	0.929	0.727–1.187	0.556
Death	0.828	0.353–1.940	0.663
Prolonged length of stay	1.001	0.890–1.126	0.986
Prolonged operation time	1.624	1.492–1.768	**< 0.001**
Unplanned reoperation	0.871	0.641–1.183	0.376
Unplanned readmission	0.851	0.688–1.051	0.134

*Odds ratios could not be reported due to limited number of cases

## Discussion

We sought to evaluate cases of parathyroidectomy by ENTs vs. general surgeons and the influence of surgical subspecialty on postoperative outcomes in parathyroidectomy. We found that the majority of parathyroidectomy is performed by general surgeons, which aligns with previous literature. We found that 86.7% of PT were performed by general surgeons, which is higher than the previously reported 68.8% in a 2010–2016 MarketScan analysis of privately insured patients [13]. It is important to note that the NSQIP is not a nationally representative sample and is dependent on institution participation, which may explain this discrepancy.

As expected, parathyroidectomy patients have a female predilection. Previous literature has described a 3.3:1 female-to-male ratio (i.e., approximately 76.7% female percentage) for parathyroidectomy for primary hyperparathyroidism [14]. The distribution of gender across surgical subspecialty was not found to be statistically significant, and both subspecialties had a female proportion of approximately 75%. However, ENT patients had a higher proportion of 65 + years old, Hispanic, and obese patients, while general surgery patients were more likely to be diabetic, dyspneic, hypertensive, on dialysis, on steroids, and have a bleeding disorder or preoperative sepsis. While our findings on variation in race and age are corroborated by previous analyses that found no differences in case complexity between surgical subspecialty in thyroid and parathyroid operations, the notable variation in comorbidities between the cohorts in our study implies that case complexity or predisposing patient factors may not always be consistent between the two subspecialties [7].

Although thyroid and parathyroid surgery is typically performed by general surgeons in most European countries and is still predominantly performed by general surgeons in the United States, previous literature has noted that the sub-specialty itself is less contributory to surgical outcomes than training, experience, and skill [15]. Similarly, an analysis of racial disparities in thyroid and parathyroid surgeries found that high surgeon volume (> 100 cases over 7 years) is associated with nearly a seven-fold decrease in mortality when compared to low volume surgeons (< 10 cases over 7 years) [16]. The relationship between case volume and outcomes has further been supported by a recent systematic review, although most studies included in this review did not stratify outcomes based on subspecialty training [17].

Unadjusted chi square analysis indicated that ENT cases had shorter LOS, in addition to less cases of septic shock, acute renal failure, unplanned reoperation, and unplanned readmission, while general surgery cases had shorter operative times. However, after performing an adjusted logistic regression analysis, no differences in LOS persisted, though differences in operative time did. It is also important to note that no statistically significant differences in complication incidences were found. This largely agrees with a prior evaluation of the impact of surgical subspecialty on parathyroidectomy outcomes as being largely noncontributory [7]. The lack of differences in complications based on subspecialty were also similarly found in a 2012–2016 NSQIP analysis of pediatric thyroidectomies [9]. Additionally, a European retrospective analysis found no significant variation in outcomes for thyroid surgery performed by otolaryngologist as compared to general surgery with the notable exception that otolaryngologist-performed thyroidectomy has a reduced incidence of vocal cord paralysis (4.7% vs. 8.2%; *p* < 0.001) [18].

Operative times were found to be significantly different between subspecialties; we found that otolaryngologists had a significantly longer operating time, which is in accordance with past literature [7]. Differences in operative time can possibly be attributed to inherent differences in case complexity, patient anatomy, involvement of trainees, or usage of nerve monitoring which could not be accounted for in this analysis. Because the NSQIP stopped tracking resident involvement from 2015 to 2018, we included all cases of parathyroidectomy. However, 45.0% of cases from 2005 to 2014 involved residents, which is notable because previously, longer operating time in the academic setting was partly explained by a greater trend of increasing otolaryngology resident exposure to parathyroidectomy as compared to general surgery residents [19]. Between 2004 and 2008, in an evaluation of cases with resident participation, otolaryngology residents performed over twice as many endocrine cases as general surgery residents, with otolaryngology residents performing more parathyroidectomy (11.6 vs. 8.8; *p* = 0.007) than their peers in general surgery [19]. Another contributory factor in operative time variation could be different practice philosophies between ENT and general surgery. In cases of PT and thyroidectomy, differences by residency training type and surgical volume were found to be significantly correlated with surgical approach [20]. In cases of thyroidectomy, 55% of otolaryngologists surveyed reported using recurrent laryngeal nerve monitoring almost always, whereas 61% of general surgeons rarely used monitoring [20].

One unique finding in this analysis was that although otolaryngologists had longer operative time, ENT cases had a significantly lower LOS on unadjusted analysis, although this difference in length of stay did not persist on adjusted analysis. Further, while we found a significant difference in operative time, this difference was only 8 min (91 min vs. 99 min), which is unlikely to be clinically relevant. Regardless, this differs from a previous 2005–2010 NSQIP analysis comparing educational trends in head and neck surgery which did not find a statistically significant difference in LOS between surgical subspecialties [7]. Regardless, it is important to note that the lack of difference in LOS on adjusted analysis indicates that this difference may not be explained by subspecialty alone. Overall, our findings regarding a shorter general surgery operative time and no other differences in complications, including length of stay on adjusted analysis, have been corroborated by previous NSQIP analysis [7].

While our multivariate logistic regression adjusted for patient comorbidities, case complexity, such as adenoma size and location could not be adjusted for and likely influenced operative times. Other relevant limitations include the possibility of other confounders unaddressed by the measured comorbidity variables in the NSQIP, limitations typical of retrospective database analyses. Furthermore, coding errors are possible within the NSQIP. Additionally, parathyroidectomy-specific postoperative complications like vocal cord paralysis or hypocalcemia are not captured in the NSQIP database.

Our analysis of outcomes between otolaryngology and general surgery cases of parathyroidectomy found significant differences in operative time. Surgical subspecialty did not appear to impact postoperative complications or morbidity. Future investigations are warranted to determine whether subspecialty training or case complexity can account for these findings.

## Electronic Supplementary Material

Below is the link to the electronic supplementary material.

Supplementary Material 1

## Declarations

### Data Sharing

The data that supports the findings of this study is openly available in the ACS National Surgical Quality Improvement Program at https://www.facs.org/quality-programs/data-and-registries/acs-nsqip/.
