# Supplementary material for: Surgical Subspecialty and Parathyroidectomy Outcomes: A National Analysis
Source: Indian J Otolaryngol Head Neck Surg. 2025 Apr 2;77(4):1856–62. doi: 10.1007/s12070-025-05436-1 (PMC11985825; doi:10.1007/s12070-025-05436-1)
Supplement: Supplementary file 1 — Supplementary Material 1 [file 12070_2025_5436_MOESM1_ESM.docx]

| **Department of Otorhinolaryngology**  **Head & Neck Surgery**  3400 Bainbridge Avenue  Medical Arts Pavilion, 3^rd^ Floor  Bronx, New York 10467  718-920-4646 Office  718-405-9014 Fax | **Christina H. Fang, MD**  Assistant Professor  Division of Rhinology & Endoscopic Skull Base Surgery  718-920-4646 Office  718405-9014 Fax  cfang@montefiore.org |
| --- | --- |

November 23, 2024

Dr. Vikas Gupta MBBS, MS

Editor-in-Chief, *Indian Journal of Otolaryngology and Head & Neck Surgery*

Dear Dr. Gupta,

We are pleased to submit our article, “**Surgical Subspecialty and Parathyroidectomy Outcomes: A National Analysis”** to be considered for publication in *Indian Journal of Otolaryngology and Head & Neck Surgery.*

Parathyroidectomy is a common procedure performed for hyperparathyroidism, amongst other conditions. Both general surgeons and otolaryngologists can perform this endocrine surgery, despite undergoing different training pathways. Surgical subspecialty has been characterized as a factor that can, at times, contribute to variation in postoperative outcomes, though results naturally vary by specialty and by procedure. Despite this, there is limited literature on the influence of surgical subspecialty on postoperative parathyroidectomy outcomes. In our study, we utilized the National Surgery Quality Improvement Program (NSQIP) to evaluate the influence of surgical subspecialty on patient outcomes following parathyroidectomy on a national level.

None of the authors have potential conflicts of interest with material presented herein. Each of the authors contributed substantially to the creation of this work. This report is not in consideration for publication elsewhere. We look forward to your decision on our paper.

Sincerely,

**Christina H. Fang, MD**

Assistant Professor

Department of Otorhinolaryngology – Head and Neck Surgery

Montefiore Medical Center

The University Hospital of Albert Einstein College of Medicine
